# Evaluating match running performance in elite Australian football: a narrative review

**DOI:** 10.1186/s13102-021-00362-5

**Published:** 2021-10-26

**Authors:** Christopher Wing, Nicolas H. Hart, Fadi Ma’ayah, Kazunori Nosaka

**Affiliations:** 1 Centre for Exercise and Sports Science Research, School of Medical and Health Sciences, Edith Cowan University, 270 Joondalup Drive, Joondalup, WA 6027 Australia; 2 Exercise Medicine Research Institute, Edith Cowan University, Joondalup, WA Australia; 3 Institute for Health Research, University of Notre Dame Australia, Fremantle, WA Australia; 4 Caring Futures Institute, College of Nursing and Health Science, Flinders University, Adelaide, SA Australia; 5 School of Education, Curtin University, Bentley, WA Australia

**Keywords:** High-speed running, Match factors, Match-analysis, Microsensor technology, Running distances

## Abstract

During Australian football (AF) matches, players are subjected to high running loads, which are intermittent in nature. There is a growing body of research that highlights factors which can both positively and negatively affect this match running performance (e.g., the total distance travelled by a player during match-play). In order to appropriately evaluate these factors, a thorough search of MEDLINE, SportDiscus and Web of Science databases was performed, with a total of 17 manuscripts included within the final evaluation. The main findings from this review highlighted that match running performance is increased amongst those playing in midfield and half back/forward positions, in players with lower playing experience, as well as in matches against higher quality opponents, and in losing quarters. Additionally, a well-design interchange-rotation strategy may be able to positively affect match running performance. A decrease in match running performance was evident amongst more experienced players, during periods of acute fatigue (e.g., following periods of high intensity activity), during matches played in higher temperatures and matches with an increased number of stoppages. However, no effect of ground hardness or size, as well as responses to self-reported wellness questionnaires was found. Other factors such as finals series matches, pre-season training load and elements related to the schedule have been shown to have substantial conflicting results within the literature, increasing the difficulty in making generalisable conclusions to their effect on match running performance. Developing a thorough understanding of these factors which affect match running performance can aid practitioners and coaches to gain a greater understanding of a player’s performance as well as inform the development of strategies for its improvement.

## Background

Australian Football (AF) is an intermittent sport, played between two teams of 18 players plus 4 players on the interchange-bench [1]. During AF matches, players are required to transfer the ball through kicks and handballs to create a shooting opportunity [1]. At the elite level, games are played across 4 quarters of 20 min in duration plus time on (a time period added to account for all stoppages in play). Typically, this leads to matches lasting in excess of 100 min [2].

Oftentimes, wearable microsensor technology (inclusive of a global positioning system and micro-electrical–mechanical system) is utilised to ascertain the physical output of AF players during match play [1–5]. Technology of this nature is able to provide a variety of metrics concerning match running performance, including distances travelled in a variety of velocity bandings as well as accelerations and decelerations [1–5]. A recent systematic review in this area has shown that elite level players travel around 12,897 ± 1601 m during match-play, which expressed relative to playing time was reported as 129 ± 13 m·min^−1^ [1]. Information of this nature is often utilised to appropriately plan and monitor individual and team training prescription.

However, several factors may both positively or negatively impact the distances travelled by athletes during competitive matches [6, 7]. It is important for practitioners working with AF players to have a full appreciation of how, and to the extent of which, these factors can affect match running performance. This can enable a greater understanding of athletic performance, improve upon training program design and inform tactical periodisation strategies [6, 8].

Traditionally, research within this area has focused heavily upon a player’s physical capacity and its effects on match running performance, which have been well established [9, 10]. However, recent literature has brought several other factors to light, including those related to the match (e.g., match outcome) and the environment (e.g., the temperature) [6, 7]. Whilst we recognise the recent, large research study by Esmaeili et al. [6] in this area, there is a need for reviews of this kind in order to give a comprehensive overview of the available literature, whilst both strengthening our understanding, and identifying gaps within in our current knowledge.

Therefore, the aim of this narrative review is to present a thorough investigation of the current literature surrounding the range of factors that can affect match running performance (e.g., total running distances performed by players during AF matches), and provide recommendations for the use of the data within practical or applied settings. Although the information within this review focuses on AF, the findings have potential to be applied to other sports such as soccer, rugby, Gaelic football and gridiron.

## Methods

In order to achieve these aims, a comprehensive search of MEDLINE, SportDiscus and Web of Science was performed through to December 2020, to identify original research articles describing Australian Football match running performance. The following terms were included within the search which were combined using “AND”:

1. “Australian football” OR “football” OR “AFL” OR “Australian football league” OR “Australian football players” OR “Australian rules football”
2. “Movement patterns” OR “movement demands” OR “running performance: OR “game demands” OR “match performance” OR “match characteristics” OR “activity profiles” OR “locomotion” OR “match play” OR “athletic performance”
3. “Mircrosensor technology” OR “global positioning systems” OR “GPS” OR “time motion analysis” OR “GPS output” OR “accelerometry”

Manuscripts were included if they reported the effect of at least one factor on at least one measure of running performance amongst male elite level players. Male players were focused upon as the elite women’s competition (AFLW) is newly established, therefore data pertaining to this cohort is limited within the current literature and thus does not warrant review at this time. For the purposes of this review, elite level players were those considered professional and playing in the top division in Australia, the Australian Football League (AFL). Where results were reported in km h^−1^ they were converted to m s^−1^ to 1 decimal place, and both values were reported within this review. Additionally, as the effect of physical capacity has been widely reported, the focus of this review is upon the other factors, which include; playing position, training load, playing experience, fatigue, schedule, opponent, interchange-rotations, stoppages, match outcome, finals series matches, and the environment (Table 1).

**Table 1:** Summary of findings of factors that affect match running performance

Factor	Author(s)	Measure(s) of running performance	Comparison/measurement tool	Summary of findings
Playing Position	Hiscock et al. [4]	Total distance V1 distance Velocity load	Key and nomadic position	Nomadics greater activity profile than key position (*p* < 0.05)
	Coutts et al. [2]	Total distance High-speed (> 4 m s^−1^) Very-high speed (> 5.5 m s^−1^) Sprint speed (> 6.7 m s^−1^)	Tall backs/forwards, mobile backs/forwards, ruck, midfield	Generally, midfielders and mobile position players outperformed ruck and tall position players
	Ryan et al. [7]	Total distance High-speed (> 5.6 m s^−1^)	Small backs/forwards, midfield, ruck, tall forwards/backs	Minor influence on rel. distance Rel. high-speed distance ↑ small forwards/backs, ↓ ruck
	Esmaeili et al. [6]	Total distance High-speed (> 5 m s^−1^) PlayerLoad™	Nomadic, key defender, key forward, ruck	Nomadic > key defender (ES: moderate-large) Nomadic > key forward (ES: moderate) Nomadic > ruck (ES: small-large)
Training Load	Johnston et al. [12]	Total distance Low speed (0–4.9 m s^−1^) High-speed (≥ 5 m s^−1^) PlayerLoad™	High load (HTL) (365 ± 38 km) Moderate load (MTL) (307 ± 63 km) Low load (LTL) (224 ± 55 km)	Rel. distance: HTL > MTL (*p* = 0.029, ES = 0.78 ± 0.50) Rel. distance: HTL > LTL (*p* = 0.053, ES = 0.99 ± 0.51) Rel. distance: MTL = LTL (*p* = 0.998, ES = 0.34 ± 0.49) Rel. high-speed: HTL > MTL (*p* = 0.122, ES = 0.93 ± 0.50) Rel. high-speed: HTL > LTL (*p* = 0.064, ES = 1.03 ± 0.52) Rel. high-speed: MTL = LTL (*p* = 0.593, ES = 0.25 ± 0.49) Rel. PlayerLoad™: HTL < MTL (*p* = 0.04, ES = − 0.89 ± 0.46) Rel. PlayerLoad™: HTL < LTL (*p* = 0.01, ES = − 0.96 ± 0.47)
	Ryan et al. [13]	Total distance High-speed (> 5.6 m s^−1^)	Effect of Acute (7-day) and Chronic (3-week) running load	↑ 7-day total distance = ↓ rel. total distance (ES = 0.13 (0.02–0.24) ↓ 3-week average total distance = ↓ rel. high-speed distance (ES = 0.14 (0.03–0.25) No effect of pre-season training completion
Playing Experience	Hiscock et al. [4]	Total distance V1 distance Velocity load	Three experience groups (1–3, 4–6, ≥ 7 years)	≥ 7 years’ experience = ↓ movement profiles (*p* < 0.05) than 1–3- and 4–6-year players Rel. distance and V1 distance: 4–6 years < 1–3-year players (*p* < 0.05)
	Black et al. [14]	Low-speed (0–2.78 m s^−1^) Moderate-speed (2.79–4.14 m s^−1^) High-speed (≥ 4.15 m s^−1^)	Mean, peak and subsequent 3-min time period per quarter Experience classification: Experienced ≥ 5 years Less experienced ≤ 4 years	No differences in any metric for peak periods Rel. high speed: experienced ↑ than less experienced in subsequent periods of quarter 2 (ES = 0.42 ± 0.3), and quarter 3 (ES = 0.38 ± 0.33)
	Esmaeili et al. [6]	Total distance High-speed (> 5 m s^−1^) PlayerLoad™	Experience classification: 1–2, 3–6 and 7 + years	1–2 versus 3–6 years = ↑ rel. total distance (2.8% (1.5–4.0)) and PlayerLoad (4.5% (2.2–6.8)) 3–6 versus 7 + years = ↑ rel. distance (2.2% (1.2–3.2))
Fatigue	Ryan et al. [13]	Total distance High-speed (> 5.6 m s^−1^)	Questionnaire responses (muscle soreness, sleep, fatigue, stress, and mood)	No effect on match running performance
	Bellinger et al. [21]	Total distance High-speed (> 6.7 m s^−1^) PlayerLoad™	Subjective wellness (mood, energy, stress, leg heaviness, muscle soreness, sleep quality, hours slept)	No significant effect
	Mooney et al. [18]	High-speed (> 4.2 m s^−1^) Accelerometer load	Flight time: contact time (FT:CT) from jump testing	High-speed running performance was maintained regardless of fatigue state
	Cormack et al. [19]	High-speed (> 4.2 m s^−1^) Accelerometer load	Flight time: contact time (FT:CT) from jump testing	Fatigue ↓ vertical component of the accelerometer load and lead to ↑ running at low speed
	Esmaeili et al. [6]	Total distance High-speed (> 5 m s^−1^) PlayerLoad™	Interchange stints during a quarter, first versus subsequent	Subsequent stint = ↓ rel. distance ( − 5.4% ( − 5.6 to − 5.2)), high-speed distance (20.5% ( − 21.3 to − 19.8)), and PlayerLoad™ ( − 6.6% ( − 6.8 to − 6.4)
	Dillon et al. [20]	Total distance High-speed (> 5.6 m s^−1^)	Accumulated distance on subsequent rotation bout	Accumulated distance ↓ rel. total (ES = 0.17) and rel. high-speed (ES = 0.13) distances Accumulated high speed distance ↑ rel. high-speed (ES = 0.12)
	Black et al. [14]	Low-speed (0–2.78 m s^−1^) Moderate-speed (2.79–4.14 m s^−1^) High-speed (≥ 4.15 m s^−1^)	Peak and subsequent 3-min time period per quarter	All measures reduced in the subsequent period following short bout of high intensity activity
Schedule	Hiscock et al. [4]	Total distance V1 distance Velocity load	Home versus away Days between games Day versus night	Rel. V1 distance: ↑ in away games (*p* < 0.05) Rel. distance ↓ with 6- and 8-day turnaround versus 12 days Rel. velocity load: ↓ with 7-day turnaround versus 8 days No effect/ differences of day versus night
	Kempton et al. [23]	Total distance High-speed (> 4 m s^−1^) Very-high speed (> 5.5 m s^−1^) Sprint-speed (> 6.4 m s^−1^)	Comparison between start, middle and end of season	Small ↑ in running performance seen at the end of the season
	Ryan et al. [7]	Total distance High-speed (> 5.6 m s^−1^)	Home versus away Start versus end of season Days between games (short = 6 days, long = ≥ 7 days)	Rel. distance and high-speed distance ↓ in away matches Rel. distance ↓ at start of season No effect of days between games
	Esmaeili et al. [6]	Total distance High-speed (> 5 m s^−1^) PlayerLoad™	Travel for current match Travel for previous match Number of days between games	No substantial effects
Opponent	Ryan et al. [7]	Total distance High-speed (> 5.6 m s^−1^)	Effect of strong opposition (rank 1–6)	Rel. distance ↑ against strong opposition
Interchange-rotations	Dillon et al. [20]	Total distance High-speed (> 5.6 m s^−1^)	Number of rotations in quarter Previous rotation duration Rotation duration	↑ Number of rotations = ↓ in rel. total (ES = 0.24) and high-speed (ES = 0.18) distances ↑ previous duration = ↓ in rel. total (ES = 0.09) and high-speed (ES = 0.05) distances ↑ duration of rotation = ↓ in rel. total (ES = 0.33) and high-speed (ES = 0.10) distances
	Ryan et al. [7]	Total distance High-speed (> 5.6 m s^−1^)	Number of rotations	↑ In number of rotations = ↓ in rel. total and high-speed distance
	Montgomery and Wisbey [29]	Total distance	Number of rotations Duration of rotation	↓ In number of rotations per game = ↓ in rel. distance ↑ In on-field rotation time = ↓ in rel. distance
	Mooney et al. [28]	Total distance Low-speed (< 4.2 m s^−1^) High-speed (> 4.2 m s^−1^) % High-speed Accelerometer load	Number of rotations	↑ Rotations = ↑ in rel. total, high and % high-speed distances, and accelerometer load but not low speed distances
	Esmaeili et al. [6]	Total distance High-speed (> 5 m s^−1^) PlayerLoad™	Stint duration Recovery duration	↑ Stint duration (+ 14 min) = ↓ in rel. total ( − 6.4% ( − 6.6 to − 6.2)), high-speed distances ( − 12.4% ( − 13.4 to − 11.4%)), and PlayerLoad™ ( − 7.7% ( − 7.8 to − 7.6)) ↑ recovery time (+ 4 min) = ↑ rel. total (1.0% (0.7 – 1.2)), high-speed distances (6.7% (5.6–7.9)), and PlayerLoad™ (1.2 (0.9–1.5))
Stoppages	Dillon et al. [20]	Total distance High-speed (> 5.6 m s^−1^)	Number of stoppages	↑ Stoppages = ↓ in rel. total (ES = 0.17) and high-speed (ES = 0.05) distances
	Ryan et al. [7]	Total distance High-speed (> 5.6 m s^−1^)	Number of stoppages	↑ Stoppages = ↓ in rel. total distances, but no effect on high-speed distances
Match Outcome	Hiscock et al. [4]	Total Distance V1 distance Velocity Load	Quarters won versus quarters lost Score margin	Movement profiles slight ↑ when quarter lost (non-significant) Rel. distance: inverse relationship with margin (r = − 0.25, *p* < 0.05)
	Ryan et al. [7]	Total distance High-speed (> 5.6 m s^−1^)	Winning versus losing games	Matches won = ↑ rel. total distance but ↓ high-speed distance
	Sullivan et al. [30]	Total distance High-speed (> 4 m s^−1^) BodyLoad™ Peak Speed Accelerations (0–4 m s^−1^)	Quarters won versus quarters lost Margin of quarters won and lost (small < 9 points, moderate 8–10 points, large > 19 points)	Quarters lost = ↑ rel. high speed (*p* = < 0.001), sprints (*p* = 0.006), and peak speed (*p* = 0.008) Large margin = ↓ rel. distance (*p* < 0.001), high-speed (*p* < 0.005), and BodyLoad (*p* = 0.031)
	Esmaeili et al. [6]	Total distance High-speed (> 5 m s^−1^) PlayerLoad™	Match outcome and score margin	No substantial effect
Final’s series	Aughey [33]	Total distance High-speed (4.17–10 m s^−1^) Accelerations (2.78–10 m s^−2^)	Regular season versus finals series games	Final’s series = ↑ rel. total (11%, ES = 0.78 ± 0.30), high-speed (9%, ES = 0.29 ± 0.25), and accelerations (97%, ES = 1.30 ± 0.20)
	Esmaeili et al. [6]	Total distance High-speed (> 5 m s^−1^) PlayerLoad™	Regular season versus finals series games	Final’s series = ↓ rel. total ( − 1.7% ( − 3.3 to 0)), high-speed distances ( − 9.9% ( − 14.8 to − 4.8), and PlayerLoad™ ( − 2.5% ( − 4.6 to − 0.4))
Environment	Hiscock et al. [4]	Total distance V1 distance Velocity Load	Wet versus dry	Rel. velocity load: ↑ in wet games
	Aughey et al. [39]	Total distance High-speed (4.17–10 m s^−1^) Sprints (> 6.94 m s^−1^) Accelerations (> 2.78 m s^−2^)	Hot versus cold matches	Hot matches = ↓ rel. total distance but preserved rel. high-speed, sprints and accelerations
	Esmaeili et al. [6]	Total distance High-speed (> 5 m s^−1^) PlayerLoad™	Light rain (≤ 1 mm) versus dry Moderate rain (> 1 mm) versus dry Apparent temperature (+ 12 °C) Ground hardness (+ 17 gravities) Ground size (+ 6,600 m^2^)	No substantial effect of light rain Moderate rain = ↓ rel. total ( − 2.2% ( − 5.6 to 1.3)) and high-speed distances ( − 9.2% ( − 19 to 1.9)) ↑ Temperature = ↓ rel. total ( − 2.0% ( − 2.6 to − 1.3)) and high-speed distances ( − 6.1% ( − 8.3 to − 3.9)) No substantial effect of ground hardness No substantial effect of ground size

Key; VI distance: distance above an individual’s aerobic threshold; Rel, relative. Speeds have all been converted from km·h^−1^ to m·s^−1^ where necessary

## Main body

The 17 manuscripts highlighted 11 factors which should be considered when evaluating match running performance. These included playing position, measures of training load (during both pre and in-season phases), playing experience, fatigue (acute and chronic), schedule (stage of season, home and away games, rest between games), opponent (high versus low quality), inter-change rotations (length, amount and bench time), stoppages, match outcome (both result and score margin), finals series matches and the environment (temperature, rainfall, ground hardness and oval size). Each of these factors has been explored to asses if they have either a positive (e.g., an increase), negative (e.g., a decrease) or no effect on one or more measures of match running performance (e.g., total running distance).

### Playing position

**Fig. 1:**
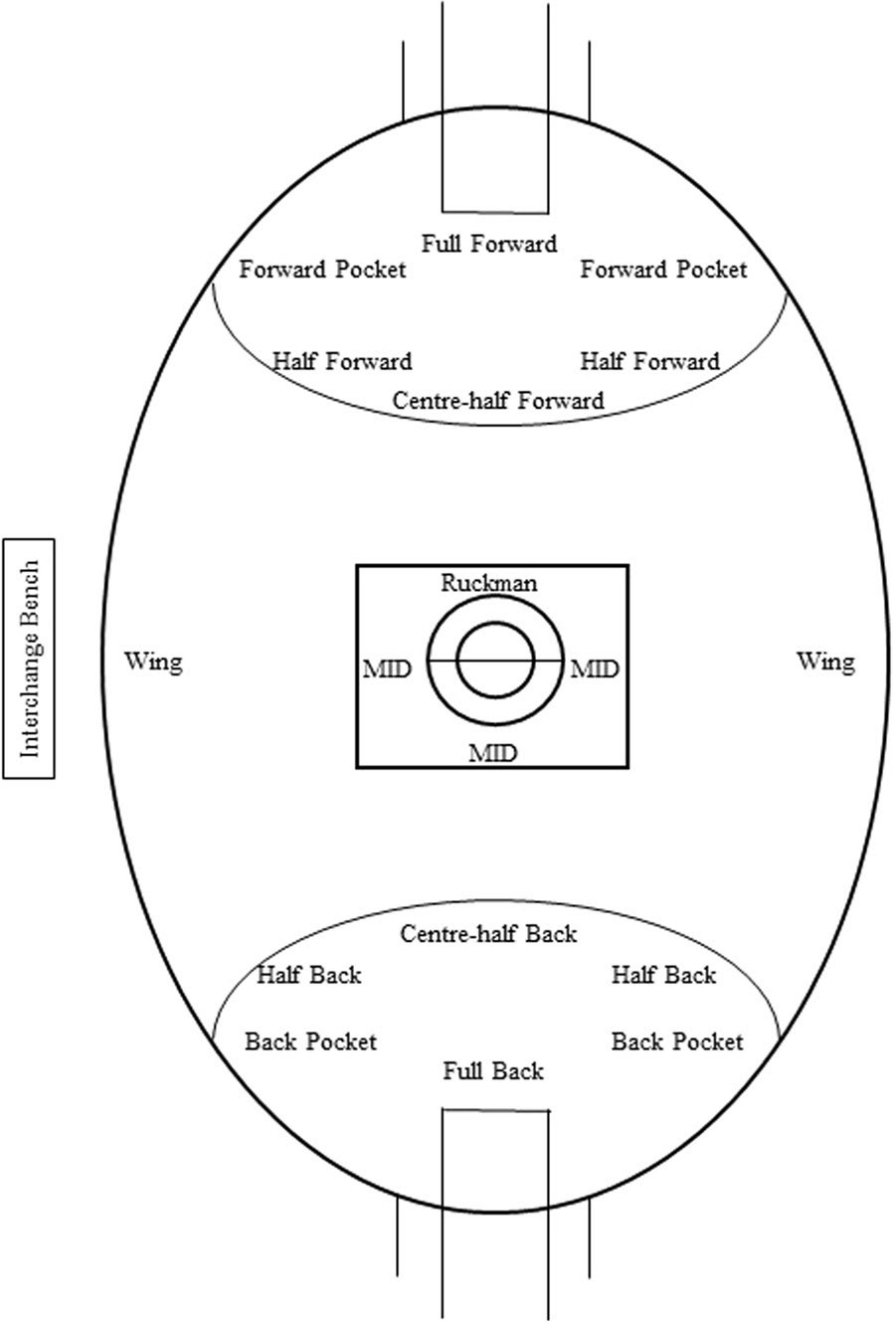
Playing oval and playing positions. Key; MID: Midfield

Oftentimes, match running performance is described with players delineated into various playing positions (Fig. 1). This practice is often complicated by sample sizes, which can prevent the analysis of players in discrete groups, with players often grouped into more general positions (e.g., nomadics, key position). In this instance, nomadic players (typically midfielders and half line players) have been shown in one study to have greater (*p* < 0.05) movement demands than key position players [4], and in a second to complete more distance, high-speed distance and PlayerLoad™ than key defenders (effect sizes: moderate-large), key forwards (effect size: moderate) and the ruck position (effect size: small to large) [6].

Coutts et al. [2] were able to divide players into specific playing groups, demonstrating that midfielders and mobile backs performed the greatest distances, with tall forwards performing the least. Additionally, midfielders performed significantly more high-speed distance than all other positions, with mobile backs and forwards outperforming tall backs and forwards, as well as the ruck position [2]. However, more recent research has found that playing position only had a minor influence on relative distance, with a greater effect noted on relative high-speed running, which was highest amongst the small forwards and backs, and lowest for the ruck position [7]. This may be indicative of the evolution of the game, where players are often required to play in multiple positions in one game, which has the potential to distort findings related to playing position [7]. Nonetheless, the evidence presented leans towards greater demands being placed upon the smaller position players (e.g., midfielders, half line players, nomadics), with lower demands experienced by taller position players (ruck, key position, full back/forward).

### Training load

It is common practice within team sports, such as AF, to monitor athlete training load during both the pre and in-season phases [11]. Johnston et al. [12], studied the pre-season training loads of 44 elite male AF players divided into 3 training load groups, based upon total running distance recorded during pre-season; high load (365 ± 38 km), moderate load (307 ± 63 km) and low load (224 ± 55 km) [12]. The match activity profiles demonstrated that the high load group performed more relative total and high-speed (≥ 5 m s^−1^) running distances than both the moderate (*p* = 0.029, ES = moderate: 0.78 ± 0.50; *p* = 0.122, ES = moderate: 0.93 ± 0.50) and low training load groups (*p* = 0.053, ES = moderate: 0.99 ± 0.51, *p* = 0.064, ES = moderate: 1.03 ± 0.52) respectively [12]. Additionally, pre-season high-speed running load was a significant predictor for match relative high-speed (*r* = 0.561 *p* = 0.001) and relative total (*r* = 0.417, *p* = 0.001) running distances, with these associations reported to be greater within the high load training group [12]. However, Ryan et al. [13] report contradictory findings, with no effect of pre-season training completion upon match running performance. This difference may be owed to the differing methodologies, where Ryan et al. [13] only reported the proportion of pre-season completion, which does not give an indication to the precise meterage of running loads completed. Furthermore, dividing players into three training load groups, as in the research by Johnston et al. [12], could be questioned. Training load prescription and management is often individual in nature in order to accurately balance fitness and fatigue, and to reduce the chance of injury [11]. Therefore, applying a global volume of load with the aim to increase match running performance, as implied by the findings of Johnston et al. [12], may be problematic in practical settings. Additionally, training load prescription is also often tailored to both an individual’s physical capacity and the requirements of their role within the team, which could further explain this finding [12].

Ryan et al. [13] established that an increase in 7-day total running distances, and decreases in 3-week average total running distances during the season was associated with reduced relative total distance and relative high-speed running distances respectively during competitive matches. Although this finding may suggest that a balance must be struck when prescribing training volumes in-season [13], it should be noted that the effect sizes were small (0.13 and 0.14 respectively).

### Playing experience

Hiscock et al. [4] reported that as playing experience increased, match activity decreased [4]. Specifically, those with 7+ years’ experience recorded lower (*p* < 0.05) physical match output than both 1–3- and 4–6-year players, with the 4–6-year players recording lower (*p* < 0.05) relative and V1 (distance above aerobic threshold) running distances compared to the 1–3-year players [4]. In support of this finding, a second study found that the less experienced players performed greater relative total distances than their experienced counterparts, but not relative high-speed distances, which remained constant across experience groups [6]. The authors speculate that these differences may be owed to the greater positioning and reading of the game of the more experienced players, thus reducing their movement requirements [4].

Black et al. [14] studied the effect of playing experience on elite AF players ability to perform during peak and subsequent 3-min periods of each quarter, with contrasting results to the aforementioned study. They found that the experienced players (≥ 5 years playing experience) performed more high-speed (≥ 4.15 m s^−1^) running per minute in the subsequent periods of quarters 2 and 3 than the less experience players (≥ 75% likelihood of the smallest worthwhile difference) [14]. However, no meaningful differences between experience groups were found concerning total running distance per minute in any subsequent periods, or for any metric during the peak 3-min periods [14]. It is speculated by the authors that these results may be subject to several contributing factors. This included playing at a higher level more frequently, which exposes the more experienced players to greater intensity match play, thus enabling them to develop the required physical attributes to cope with periods of high-intense activity [14]. This is somewhat supported within the literature where it has been reported that match activities are higher in elite level games compared to those played at the sub-elite level [15, 16]. Furthermore, the authors propose that the more experienced players may be better equipped to manage themselves through a game utilising a more effective pacing strategy, while additionally suggesting that their playing time is better managed through the use of player rotations [14]. However, the role that playing position may play in the differences between experienced and non-experienced players was not explicitly evaluated, which may have influenced the findings, particularly if players were required to play multiple positions during a match.

Together these papers provide useful information concerning the potential management of players during competitive match play. As it appears that the less experienced players are able to cover greater total match distances [4], with the more experienced players able to buffer higher intensity periods of play [14], it would appear reasonable that the more experienced players are used in shorter/ more frequent rotation periods. This would lead to a reduction in total running volume whilst allowing them to be utilised as “impact” players during highly intense or critical game moments [1].

### Fatigue

Fatigue can be classified as either chronic (impairment of performance due to prolonged exposure to high running loads) or acute (a sudden inability to maintain output during competition because of exertion) [17]. Previous AF research has made use of the flight time: contraction time (FT:CT) ratio, derived from countermovement jump performance, to measure neuromuscular fatigue [18, 19]. Following an initial 4-week assessment to establish baseline measures, FT:CT measures were taken throughout an entire AF season [18, 19]. Cormack et al. [19] established that neuromuscular fatigue lead to a reduction in the vertical vector component of the accelerometer, and that there was a tendency for players to perform more low speed running and less acceleration efforts.

Utilising the same methodology, however, Mooney et al. [18] found that neuromuscular fatigue had no effect on high-speed running distances when corrected for yo-yo intermittent recovery test performance. This finding may be partly attributed to 4.2 m s^−1^ (15 km h^−1^) being used to categorise high-speed running [18]. This appears to be particularly low when compared to other speeds utilised to define this speed banding within AF practices [7, 20]. It is therefore speculated by the authors that neuromuscular fatigue may have played a greater role in reducing high-speed running loads had the velocity band been set higher (e.g., > 6.7 m s^−1^ (> 24 km h^−1^)) [18].

Pre-match subjective wellness scores, that are widely used as surrogate measures of fatigue and recovery, do not appear to have a substantial effect on match running performance. The reports by both Bellinger et al. [21] and Ryan et al. [13] made use of pre-match wellness questionnaires, which included measures of mood, stress, fatigue, muscle soreness, sleep, energy and leg heaviness, with no significant effects found upon match running performance. In the case of Ryan et al. [13], the authors acknowledge that questionnaires were carried out 3–4 days pre-match, which may have limited their applicability in measuring subjective wellness in relation to match running performance. However, Bellinger et al. [21] were still unable to find a significant effect, even when questionnaires were carried out pre-game on match day, therefore questioning the role of wellness questionnaires when predicting match running performance.

The role of acute fatigue upon running performance has also been reported within the literature [14, 20]. Previous research established that total, moderate (2.79–4.14 m s^−1^) and high-speed (≥ 4.15 m s^−1^) running distances per minute were all reduced (≥ 75% chance of effect being greater than smallest worthwhile change) following peak periods of high intensity activity [14]. Despite this, it should be noted that the match context may have had an impact on the period of play following the peak intensity period. It is possible that players were still capable of high physical outputs, and were therefore not fatigued, but the game situation did not demand a high physical output (e.g., a period of play with several stoppages). Additionally, it has also been established that during short periods of activity, physical demands are reduced when technical actions (e.g., kicks) are increased, which may also contribute to this finding [22]. However, it has also been demonstrated that accumulated match running distances (i.e., distance accumulated in a quarter prior to a rotation bout) resulted in a reduction in both relative total and high-speed running distances [6, 20]. This information concerning the effects of acute fatigue upon running performance is important for AF coaches when considering an interchange rotation strategy.

### Schedule

Competitive schedules including venue (home or away), days between matches, time of the season and bouncedown time (day or night), have all been assessed in respect to their impact upon match running performance [4, 7, 23]. When studying home versus away matches, Ryan et al. [7] found that matches played away (defined as those outside of Victoria, a south-eastern state of Australia) displayed reduced relative total and high-speed (> 5.6 m s^−1^ (> 20 km h^−1^)) running distances (effect sizes: moderate to small respectively). Conversely, Hiscock et al. [4] found relative V1 (distance above individual aerobic threshold) distances to be higher in away games (41 ± 9 m min^−1^) compared to home games (38 ± 11 m min^−1^). Ryan et al. [7] highlight the potential effects of both opposition home ground familiarity and interstate-travel on the reduction in running activity, factors which have both been previously highlighted to reduce team success in AF [24]. Although the players in the study by Hiscock et al. [4] were also subjected to interstate travel, this team were based in Western Australia, where interstate travel is more frequent for these players due to the distribution of the 18 teams within the AFL competition, where only 2 are based in Western Australia, with 10 located within Victoria. While speculative, it is possible these players have developed better coping strategies and are more used to travel of this type in comparison to teams based within Victoria. This is somewhat supported in the recent literature, where it was reported that travelling for the current and previous game to have no substantial effect upon match running performance [6].

Conflicting evidence surrounding the number of days turnaround (i.e., days between matches) and its impact on match running performance exists. Previous research has reported that days between matches (short =  ≤ 6 days, long =  ≥ 7 days) to have no significant effect upon relative total or high-speed running distances [6, 7]. As it is well recognised that measures of fatigue (saliva and countermovement jump variables) can be reduced up-to 72–96 h post AF matches [25], it is also possible that teams have appropriately titrated training loads during short turnarounds in order to promote recovery, and thus reduce fatigue, which could go some way to explaining the findings of the aforementioned studies. Conversely, Hiscock et al. [4] found that matches with a 6 (131 ± 12 m min^−1^) or 8 (129 ± 13 m min^−1^) day turnaround saw reduced relative total distances in comparison to those that followed a 12 (137 ± 12 m min^−1^) day turnaround. These differences may be based upon the time frames utilised to define the turnaround, with differences only found in the report by Hiscock et al. [4] when the number of days between matches was extended to 12 days. Additionally, only a small sample size was present within the research, with only 2 games played following a 12 day recovery period [4]. Oftentimes, a 12-day recovery period is experienced by AF teams following a bye week (i.e., a week when a team is not fixtured to play during a season). As there appears to be a reported benefit following a competitive break, it may be prudent for future research to assess the impact of number of games in sequence upon match running performance, as this may have an effect upon accumulated fatigue.

Matches played towards the end of the competitive season (e.g., final 8 weeks or rounds 17–23) have been shown to display small increases in match running performance, compared to matches completed at the start of the season [7, 23]. A potential mechanism to explain this increase in running performance towards the end of the season may be due to teams fighting for finals series places, therefore raising their intensity to match the added importance associated with these matches [7, 23]. Finally, only one study investigated the influence of bouncedown time (i.e. day or night) on match running performance, and found no significant differences between the two parameters [4].

Insufficient and conflicting evidence regarding the effect of several parameters linked to the competitive schedule upon match running performance remain. Further, several of these factors are only reported within a limited number of studies, reducing the ability to generalise the results to all AF populations. However, practitioners should be encouraged to explore many of these further, and particularly within their own setting, to determine the extent to which they may influence match running performance.

### Opponent

Only one study examined the effect of opposition quality and reported higher relative total distances with no difference in relative high-speed running (> 5.6 m s^−1^ (> 20 km h^−1^)) distances in matches played against opponents classified as high quality (defined as final ladder rank 1–6) [7]. However, the authors acknowledge the problematic approach to using the final ladder position as a means to defining the quality of an opponent with factors such as form, injury and selection potentially influencing the strength of the opposition on a match-to-match basis [7, 26]. With this in mind, it may be more beneficial to take a more fluid approach to opposition ranking through either using the rank of the opposition pre-game or the difference in rank between the two competing teams [7, 26]. However, it should be acknowledged that this method is not without its own pitfalls as ladder position is often unstable in the early rounds of the season. Furthermore, caution should be exerted when drawing conclusion from single studies, highlighting the need for additional research to be performed in this area.

### Interchange-rotations

At the elite level, player interchange-rotations are currently capped at 75 per game [27]. Oftentimes, coaches implement a specific interchange-rotation strategy in order to seek a competitive advantage, either physically or tactically [28]. Due to the growing need to develop an effective interchange-rotation strategy, several researchers have highlighted the role of rotation periods upon players physical output [6, 7, 20, 28, 29].

Evidence within the literature consistently lends support to the preservation of physical match output with the presence of frequent, short to moderate, rotation bouts [6, 7, 28, 29]. Additionally, longer rotation bouts have been reported to lead to a reduction in relative total and high-speed (> 5.6 m s^−1^ (> 20 km h^−1^)) running distances [20] as well as an overall reduction in physical output [6, 29]. Therefore, understanding the effect of bout duration appears to be attractive to coaches planning interchange-rotation strategies. In this instance, it has been highlighted that moderate reductions in physical output were present between rotation bouts lasting < 5 min and those lasting 9–11 min, with a greater reduction experienced by the forward playing group [29]. Interestingly, when compared to the intensity of rotation periods < 5 min in duration, there was a trend for an increasing negative effect on physical output for every additional two minutes of on-field time [29]. This is somewhat supported by Esmaeili et al. [6] who found small to moderate reductions in physical output when comparing a typically long to a typically short rotation bout.

In addition to on-field rotation time, an increase in the number of player rotations could also contribute to increases in several parameters of match running performance [7, 28, 29]. Specifically, Montgomery et al. [29] indicate that for every player receiving less than 6 rotations per game, a 3.6% reduction in physical output is present per rotation, with players subjected to more than 6 rotations not receiving any comparable benefit. However, the applicability of this finding to current AF practices should be questioned, as the interchange cap for the 2021 season stands at 75, compared to the unlimited number permitted at the time of the aforementioned study. It is important to note that conflicting evidence exists concerning bench duration, with one study reporting no effect [20], but a second suggesting that an increase in recovery time (> 4 min) saw an increase in match running performance [6]. These differences in results may be owed to the differing sample sizes, where one study was focused on a single team [20], whilst the other investigated all 18 AFL teams [6].

Although somewhat hampered by restrictions to rotation numbers at the elite level, the evidence here supports the adoption of a rotation strategy which employs short to moderate, frequent rotations, is likely to increase a players physical output, potentially through the delaying of the onset of fatigue [6, 7, 13, 28, 29]. Additionally, and as is highlighted by Montgomery et al. [29], gaining an understanding concerning optimal rotation length can also aid to influence representative training design.

### Stoppages

Events in an AF match such as the centre-bounce, boundary throw-ins and ball-ups are all classified as stoppages [7, 20]. During this time the game is momentarily paused while the umpire restarts play. Previous research has reported that the number of stoppages can influence the match running demands of AF players [7, 20]. Dillon et al. [20] were able to demonstrate that an increase in the number of stoppages resulted in a small reduction in total and a trivial reduction in high-speed running distances. This finding is supported by a second study, which found that as the number of stoppages increased a reduction in relative total running distance, but not relative high-speed running distances, was found [7].

These findings may be owed in part to the reduced opportunity for locomotion that occur during a stoppage. This is not only due to the players being centred closer to the ball, and are therefore required to travel less distance in order to compete for possession, but also because the ball is out of play, which further reduces the intensity demands of the game [7, 20]. Australian football teams utilising a tactical periodisation approach to training may wish to highlight those teams who play a “congested” style of football, where more stoppages are likely to occur [7, 20]. In these instances, the preparation needs may centre more firmly around collision/ contact-based training as opposed to increased running distances.

### Match outcome

Match outcome, including the final result (win or loss), score margin and successful periods of play (e.g., won quarters in a match) may all influence match running performance. It has been reported that relative distance increased in winning games, with small reductions in relative high-speed (> 5.6 m s^−1^ (> 20 km h^−1^)) distances [7]. However, others have reported that match outcome did not have a significant effect on match running performance [6]. The differences here may lie within the sample, where Ryan et al. [7] was a single team, where Esmaeili et al. [6] included all 18 AFL clubs. As there are many tactical approaches, or “styles of play”, that exist, it may be that the team in the study by Ryan et al. [7] adopted an attacking approach that required a greater physical demand, which may not be reflective of every team within the competition.

When matches were sub-divided into quarters, it was demonstrated that relative high-speed (> 4 m s^−1^ (> 14.5 km h^−1^); 37 (35.9–38.2) versus 33 (32.1–34) m min^−1^), sprints (0.18 (0.17–0.20) versus 0.16 (0.15–0.18) efforts.min^−1^), and peak speed ((7.2 (7.1–7.2) versus 7.0 (7.0–7.1) m s^−1^) (25.8 (25.5–26) versus 25.3 (25.1–25.5) km h^−1^)) were all significantly higher in quarters lost compared to those that were won [30]. This is supported in a second study which found that movement profiles were increased during losing quarters compared to winning quarters, however this did not reach statistical significance [4]. Increased activity during losing quarters may be owed to ball possession, with teams that subsequently win quarters potentially having greater possession and therefore able to dictate the pace of the game [1, 31]. This, in turn, may lead to an increase in the physical output of the team out of possession as they are forced to “chase” in order to successfully defend and recover possession [1]. However, this theory is not supported by Rennie et al. [32], who demonstrated several similarities in match running performance between attacking and defensive phases of play.

The effect of quarter score margin, small (< 9 points), moderate (10–18 points) and large (> 19 points), upon physical output has also been investigated [30]. It was found that metrics including relative total distance, high-speed distance and body load to all be lower when the score margin is higher [30]. In support of this, Hiscock et al. [4] also reported an inverse relationship between score margin and relative total running distances. Although several factors may contribute towards this, one that should be highlighted is stoppages. If more goals and/or behinds are scored (as indicated by the greater score margin) then by the nature of the game, more stoppages will be created. In-turn, and as is described within a previous section of this review, stoppages reduce the opportunity for locomotion [7, 20], and therefore may be a contributing factor to this finding.

### Finals series

The activity profiles of eight elite AF players were studied during 3 regular season games and 3 finals series games against the same opposition during the 2008 season [33]. When expressed per minute of playing time, it was reported that during finals series games players covered 11% more running distance (small to moderate effect size), 9% more high-intensity (4.17–10 m s^−1^) running distance (small effect size), and also nearly twice the amount of maximal accelerations (2.78–10 m s^−1^, large effect size) [33].

However, it should be noted that although this research indicates an increase in physical output during finals series games, it is restricted to a relatively small sample size (24 samples from both regular and finals series games), whilst also being confined to the nomadic playing position [33]. This is in contrast to more recent research, involving all 18 teams within the AFL competition, which found small reductions in physical output during finals series matches [6]. The differences in these research papers highlights the importance of caution when generalising the findings made from single study/single club design to the wider population. Additionally, it is speculated that these differences may be attributed to the evolution of the tactical/technical basis of the game, where contested possession, and therefore stoppages, has increased in recent times [6, 34].

### Environment

Exercising in the heat poses several physiological challenges, including dehydration, reduced muscle function and strength, and increased cardiovascular strain, which can result in both reduced performance and fatigue [35–38]. Research within AF has demonstrated that an increase in temperature can negatively affect match running performance [6, 39]. Aughey et al. [39] compared relative total, high-velocity (4.17–10 m s^−1^) and sprint (> 6.94 m s^−1^) running distances, as well as relative maximal accelerations (2.78 m s^−2^), performed in hot (av. temp 27 ± 2 °C) versus cold (av. temp 17 ± 4 °C) matches, as classified by the rating of risk of heat illness. Despite relative total running distance being reduced during hot games in all 4 quarters, and particularly in quarters 2 and 4 (small differences), there appeared to be a preservation of high-intensity tasks (e.g., sprinting and accelerating) [39]. It is speculated by the authors that players may have adopted a pacing strategy, by reducing the total distance they ran and thus preserved energy to sustain the performance of high-intensity efforts [39]. However, Esmaeili et al. [6] found that elevated temperatures reduced both relative total and high-speed running distances. The differing results concerning high-speed running may again be attributed to sample size, but also to methodology, where Esmaeili et al. [6] assessed temperature as apparent temperature which is a function of ambient temperature, humidity and wind speed.

Hiscock et al. [4] makes comparisons of physical output during wet and dry matches. They included 83 player files from wet matches and 272 files from dry matches and found that the weather conditions only had an effect upon velocity load (measurement of running power/momentum), which significantly (*p* < 0.05) increased during wet games [4]. This is maybe somewhat surprising, as it is a common theory that during wet matches, more stoppages and “contested football” are present, which has been previously shown to reduce the opportunity for player locomotion [7, 20]. Therefore, it may have been expected that several measures of match running performance would be negatively affected by wet conditions. However, this was not the case in the aforementioned study, which reported no differences in relative running distances (wet: 134 ± 12 vs dry: 133 ± 12 m min^−1^), and relative distances travelled above a players aerobic threshold (wet: 39 ± 11 vs dry: 39 ± 11 m min^−1^) [4].

Conversely, the research by Esmaeili et al. [6] highlighted that moderate (> 1 mm) of rain had a significant detrimental effect on relative total (− 2.2%) and high-speed (− 9.2%) running distances. However, rainfall of < 1 mm, had no significant effect. The differences seen here, both between and within studies, may highlight the importance of quantifying the amount of rainfall, as opposed to generalising matches as wet or dry, when assessing the impact upon match running performance. Finally, to the best knowledge of the authors, ground hardness and size was only investigated in the research by Esmaeili et al. [6], who found no substantial effect on match running performance.

### Limitations and future recommendations

There are several limitations to this review that should be discussed. The first is the multitude of velocities utilised to define high-speed running (Table 1), which makes cross-study comparisons particularly challenging. Although speculative, it may be possible that the choice of velocity has an impact on both the significance and magnitude of effect of some factors. Additionally, different definitions are used for several of the factors discussed within the manuscripts. For example; pre-season training load was defined in one paper by total meterage travelled, and in another by the proportion of training completed. This not only makes comparisons problematic but also reduces our ability to make generalisable conclusions. This is compounded further by the conflicting evidence surrounding many of the factors, possibly due to the aforementioned reasons. The research by Esmaeili et al. [6] followed all 18 AFL teams across an entire season, which strengthens our ability to apply their findings across a wider population. However, as conflicting evidence has been noted in single team studies, it should be highlighted that the effect of some factors may be more pertinent for some clubs. Therefore, practitioners should be encouraged to make an assessment of these within their own context to ascertain which are most important within their own practical setting.

As outlined by Ryan et al. [7], an interaction of one, or several of these factors, is likely to exist. However, this has not been thoroughly explored within the literature. For example, games in elevated heat have been shown to have a negative impact on match running performance. However, it could be speculated that the magnitude of this effect could be increased against stronger opposition or on a larger playing oval, whereas the effect may be reduced in a game involving several stoppages. Furthermore, some factors may have greater impacts on specific playing positions, such as matches lost may affect backs differently to forwards. Comparisons of this nature are not only lacking within the literature, but are particularly challenging when players are often required to perform in multiple positions during a match. As noted by Esmaeili et al. [6], the role of a team’s tactical approach (e.g., a team who plays a fast possession style versus a team who plays a more congested style), has not yet been evaluated, which may modify the relationship of several factors highlighted within this review. Finally, future studies may wish to focus on women players, as their premier competition continues to evolve and more data becomes available.

## Conclusion

The aim of this review was to identify factors which affect match running performance, and provide recommendations for the use of the data within practical or applied settings. Several factors appear to affect match running performance within AF populations. Factors including matches against high quality opponents, match quarters lost, players with lower playing experience, playing in midfield or half line positions, and the adoption of frequent, short, interchange-rotations, have seen increases in match running performance. Conversely, players with greater experience, acute fatigue (e.g., accumulated load), matches with increased stoppages and increased temperatures all appear to reduce match running performance. Additionally, there appears to be no effect of responses to self-reported wellness questionnaires, ground hardness and ground size. Despite these conclusions it should be remembered that conflicting evidence exists across the literature, this is particularly evident in factors such as pre-season training load and final’s series matches, and is likely due to the different methodological approaches and samples sizes adopted by the manuscripts.

## Data Availability

All data generated or analysed during this study are included in this published article.

## Abbreviations

AF: Australian football
AFL: Australian Football League
FT:CT: Flight time:contraction time
